# Missing data in trial‐based cost‐effectiveness analysis: An incomplete journey

**DOI:** 10.1002/hec.3654

**Published:** 2018-03-24

**Authors:** Baptiste Leurent, Manuel Gomes, James R. Carpenter

**Affiliations:** ^1^ Department of Medical Statistics London School of Hygiene and Tropical Medicine London UK; ^2^ Department of Health Services Research and Policy London School of Hygiene and Tropical Medicine London UK; ^3^ MRC Clinical Trials Unit University College London London UK

**Keywords:** cost‐effectiveness analysis, missing data, multiple imputation, randomised controlled trials, sensitivity analysis

## Abstract

Cost‐effectiveness analyses (CEA) conducted alongside randomised trials provide key evidence for informing healthcare decision making, but missing data pose substantive challenges. Recently, there have been a number of developments in methods and guidelines addressing missing data in trials. However, it is unclear whether these developments have permeated CEA practice. This paper critically reviews the extent of and methods used to address missing data in recently published trial‐based CEA.

Issues of the Health Technology Assessment journal from 2013 to 2015 were searched. Fifty‐two eligible studies were identified. Missing data were very common; the median proportion of trial participants with complete cost‐effectiveness data was 63% (interquartile range: 47%–81%). The most common approach for the primary analysis was to restrict analysis to those with complete data (43%), followed by multiple imputation (30%). Half of the studies conducted some sort of sensitivity analyses, but only 2 (4%) considered possible departures from the missing‐at‐random assumption.

Further improvements are needed to address missing data in cost‐effectiveness analyses conducted alongside randomised trials. These should focus on limiting the extent of missing data, choosing an appropriate method for the primary analysis that is valid under contextually plausible assumptions, and conducting sensitivity analyses to departures from the missing‐at‐random assumption.

## 1 INTRODUCTION

Cost‐effectiveness analyses (CEA) conducted alongside randomised controlled trials are an important source of information for health commissioners and decision makers. However, clinical trials rarely succeed in collecting all the intended information (Bell, Fiero, Horton, & Hsu, 2014), and inappropriate handling of the resulting missing data can lead to misleading inferences (Little et al., 2012). This issue is particularly pronounced in CEA because these usually rely on collecting rich, longitudinal information from participants, such as their use of healthcare services (e.g., Client Service Receipt Inventory; Beecham & Knapp, 2001) and their health‐related quality of life (e.g., EQ‐5D‐3L; Brooks, 1996).

Several guidelines have been published in recent years on the issue of missing data in clinical trials (National Research Council, 2010; Committee for Medicinal Products for Human Use (CHMP), 2011; Burzykowski et al., 2010; Carpenter & Kenward, 2007) and for CEA in particular (Briggs, Clark, Wolstenholme, & Clarke, 2003; Burton, Billingham, & Bryan, 2007; Faria, Gomes, Epstein, & White, 2014; Manca & Palmer, 2005; Marshall, Billingham, & Bryan, 2009). Key recommendations include:

- taking practical steps to limit the number of missing observations;
- avoiding methods whose validity rests on contextually implausible assumptions, and using methods that incorporate all available information under reasonable assumptions; and
- assessing the sensitivity of the results to departures from these assumptions.

In particular, following Rubin's taxonomy of missing data mechanisms (Little & Rubin, 2002), methods valid under a missing‐at‐random (MAR) assumption (i.e., when, given the observed data, missingness does not depend on the unseen values) appear more plausible than the more restrictive assumption of missing completely at random, where missingness is assumed to be entirely independent of the variables of interest. Because we cannot exclude the possibility that the missingness may depend on unobserved values (missing not at random [MNAR]), an assessment of the robustness of the conclusions to alternative missing data assumptions should also be undertaken.

Noble and colleagues (Noble, Hollingworth, & Tilling, 2012) have previously reviewed how missing resource use data were addressed in trial‐based CEA. They found that practice fell markedly short of recommendations in several aspects. In particular, that reporting was usually poor and that complete‐case analysis was the most common approach. However, missing data research is a rapidly evolving area, and several of the key guidelines were published after that review. We therefore aimed to review how missing cost‐effectiveness data were addressed in recent trial‐based CEA.

We reviewed studies published in the National Institute for Health Research Health Technology Assessment (HTA) journal, as it provides an ideal source for assessing whether recommendations have permeated CEA practice. These reports give substantially more information than a typical medical journal article, allowing authors the space to clearly describe the issues raised by missing data in their study and the methods they used to address these. Our primary objectives were to determine the extent of missing data, how these were addressed in the analysis, and whether sensitivity analyses to different missing data assumptions were performed. We also provide a critical review of our findings and recommendations to improve practice.

## 2 METHODS

The PubMed database was used to identify all trial‐based CEA published in HTA between the January 1, 2013, and December 31, 2015. We combined search terms such as “randomised,” “trial,” “cost,” or “economic” to capture relevant articles (see Appendix A.1 for details of the search strategy). The full reports of these articles were downloaded then screened for eligibility by excluding all studies that were pilot or feasibility studies; reported costs and effects separately (e.g., cost‐consequence analysis); or did not report a within‐trial CEA.

For each included study, we extracted key information about the study and the analysis to answer our primary research questions. A detailed definition of each indicator extracted is provided in Appendix B. In a second stage, we drew on published guidelines and our experience to derive a list of recommendations to address missing data, and then re‐reviewed the studies to assess to which extent they followed these recommendations (see Appendix B for further details).

Data analysis was conducted with Stata version 15 (StataCorp, 2017). The data from this review are available on request (Leurent, Gomes, & Carpenter, 2017).

## 3 RESULTS

### 3.1 Included studies

**Figure 1:**
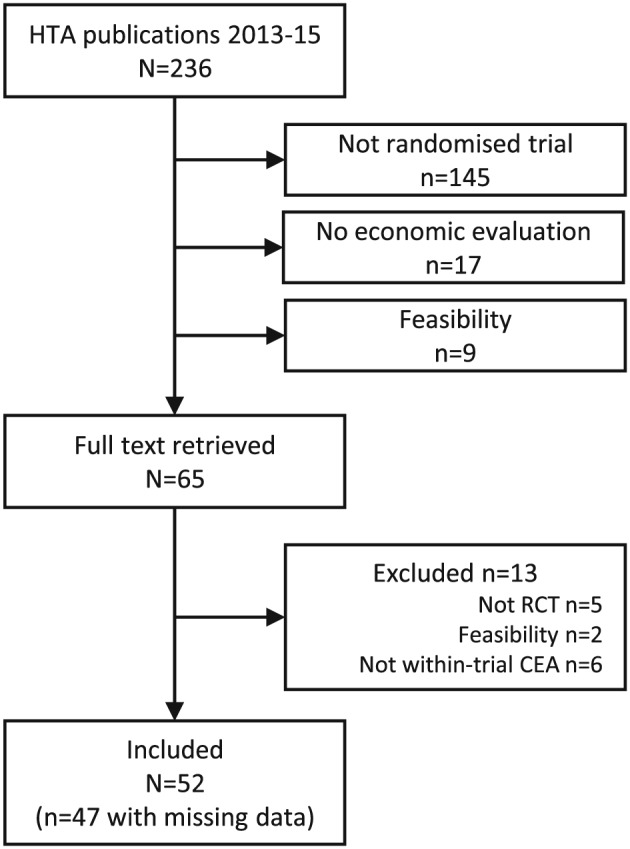
Studies selection flow diagram. CEA = cost‐effectiveness analyses; HTA = health technology assessment; RCT = randomised controlled trial

Sixty‐five articles were identified in our search (Figure 1), and 52 eligible studies were included in the review (listed in Appendix A.2). The median time frame for the CEA was over 12 months, and the majority of trials (71%, *n* = 37) conducted a follow‐up with repeated assessments over time (median of 2; Table 1). The most common effectiveness measure was the quality‐adjusted life year (81%, *n* = 42). Other outcomes included score on clinical measures, or dichotomous outcomes such as “smoking status”.

**Table 1:** Characteristics of included studies (*n* = 52)

	*n*	%
	Median	(IQR)
**General characteristics**		
Publication year		
2013	14	27
2014	15	29
2015	23	44
CEA time frame		
0–11 months	22	42
12 months	19	37
≥24 months	11	21
Follow‐up design		
Continuous (time to event)	4	8
One follow‐up assessment	11	21
Repeated assessments	37	71
Effectiveness measure		
QALY	42	81
Binary	6	12
Clinical scale score	3	6
Time to recovery	1	2
**Missing data**		
Report exact number of complete cases	20	38
Proportion of complete cases^a^	0.63	(0.47–0.81)
Proportion complete effectiveness data (*n* = 47)	0.73	(0.55–0.86)
Proportion complete cost data (*n* = 40)	0.79	(0.67–0.92)
Differs between costs and effectiveness^b^		
Yes, more cost data missing	3	6
Yes, more effect data missing	10	19
No	22	42
No missing (<5%)	5	10
Unclear	12	23
Differs between arms^c^		
Yes	10	19
No	32	62
No missing (<5%)	5	10
Unclear	5	10

*Note*. IQR = interquartile range; QALY = quality‐adjusted life year.

a Proportion of trial participants with complete cost‐effectiveness data. An upper bound was used if exact number not reported.

b More than 5% difference in the proportion of participants with complete cost or effectiveness data.

c More than 5% difference in the proportion of complete cases between arms.

### 3.2 Extent of missing data

**Figure 2:**
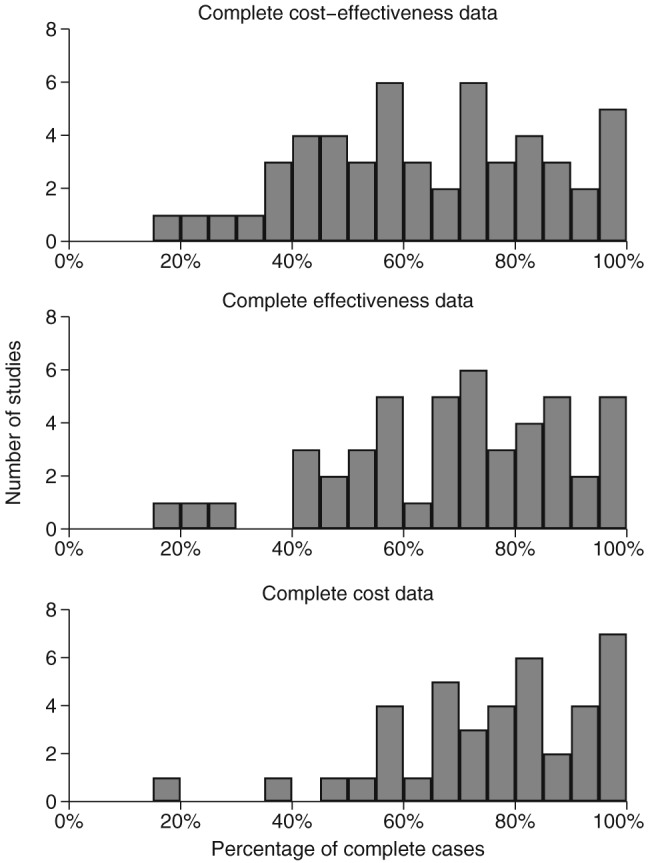
Proportion of trial participants with complete data for the primary cost‐effectiveness analysis. Shown for cost‐effectiveness (n = 52), effectiveness (n = 47, unclear in 5 studies), and cost data (n = 40, unclear in 12 studies)

Missing data was an issue in almost all studies, with only five studies (10%) having less than 5% of participants with missing data. The median proportion of complete cases was 63% (interquartile range, 47–81%; Figure 2). Missing data arose mostly from patient‐reported (e.g., resource use and quality of life) questionnaires. The extent of missing data was generally similar for cost and effectiveness data, but 10 (19%) studies had more missing data in the latter (Table 1). The proportion of complete cases reduced, as the number of follow‐up assessments increased (Spearman's rank correlation coefficient ρ = −0.59, *p* value < .001) and as the study duration increased (ρ = −0.29, *p* = .04).

### 3.3 Approach to missing data

In the remaining assessments, we excluded the five studies with over 95% of complete cases. Three main approaches to missing data were used: complete‐case analysis (CCA; Faria et al., 2014), reported in 66% of studies (*n* = 31), multiple imputation (MI; Rubin, 1987; 49%, *n* = 23), and ad hoc hybrid methods (17%, *n* = 8). For the primary analysis, CCA was the most commonly used method (43%, *n* = 20), followed by MI (30%, *n* = 14; Table 2). MI was more common when the proportion of missing data was high and when there were multiple follow‐up assessments (see Table 3).

**Table 2:** Methods for handling missing data in primary analysis (*n* = 47)

Primary analysis method	*n*	%
Complete‐case analysis	20	43
Multiple imputation	14	30
Other—single methods		
Inverse probability weighting	1	2
Bayesian model, missing data as unknown parameter	1	2
Other—ad hoc hybrid methods^a^	8	17
Using a combination of		
Mean imputation^b^	6	
Regression imputation^c^	3	
Inverse probability weighting^d^	2	
Assuming failure when outcome missing	2	
Multiple imputation	1	
Last observation carried forward	1	
Unclear	3	6

a Ad hoc hybrid method = several approaches to missing data combined, for example, using mean imputation for missing individual resource use items and multiple imputation for fully incomplete observations.

b Mean imputation = replacing missing values by the average across other participants.

c Regression imputation = replace missing values by predicted value based on observed variables.

d Inverse probability weighting = analysing complete data, weighted according to their modelled probability of being observed. These methods are presented in more details in other references (Baio & Leurent, 2016; Faria et al., 2014).

**Table 3:** Approaches to missing data, by year, number of follow‐ups, and extent of missing data (*n* = 47)

	Primary analysis method						Reported a sensitivity analysis			
	CCA		MI		Other		Yes		No	
	*n*	%	*n*	%	*n*	%	*n*	%	*n*	%
Publication year										
2013 (*n* = 13)	6	46	3	23	4	31	5	38	8	62
2014 (*n* = 15)	9	60	1	7	5	33	6	40	9	60
2015 (*n* = 19)	5	26	10	53	4	21	11	58	8	42
Number of follow‐up assessments^a^										
1 (*n* = 10)	7	70	1	10	2	20	3	30	7	70
≥2 (*n* = 36)	13	36	13	36	10	28	18	50	18	50
Proportion of complete cases^b^										
<50% (*n* = 15)	4	27	6	40	5	33	8	53	7	47
50–75% (*n* = 18)	10	56	4	22	4	22	9	50	9	50
75%–95% (n = 14)	6	43	4	29	4	29	5	36	9	64
Information missing^c^										
Similar (*n* = 22)	13	59	6	27	3	14	10	45	12	55
More cost missing (*n* = 3)	1	33	2	67	0	0	2	67	1	33
More effect missing (*n* = 10)	4	40	2	20	4	40	6	60	4	40

*Note*. % = row percentages. CCA = complete‐case analysis; MI = multiple imputation.

a Excluding one study with continuous follow‐up (*n* = 46).

b For the five studies with less than 5% of incomplete cases, four used CCA and one an ad hoc hybrid method for their primary analysis. One of the five studies conducted a sensitivity analysis to missing data.

c Excluding 12 studies where this was unclear (*n* = 35).

### 3.4 Sensitivity analyses

Over half of the studies (53%, *n* = 25) did not conduct any sensitivity analysis around missing data, with 21% (*n* = 10) reporting CCA results alone and 11% (*n* = 5) MI results under MAR alone (Table 4). The remaining studies (*n* = 22, 47%) assessed the sensitivity of their primary analysis results to other approaches for the missing data. This was usually performing either MI under MAR, or CCA, when the other approach was used in the primary analysis. Other sensitivity analyses included using last observation carried forward or regression imputation.

**Table 4:** Sensitivity analysis, overall, and by primary analysis method (*n* = 47)

	None		Sensitivity analysis method							
			CCA		MI (MAR)		MNAR		Other^a^	
	*n*	%	*n*	%	*n*	%	*n*	%	*n*	%
**Overall**										
Total (*n* = 47)	25	53	11	23	9	19	2	4	5	11
**By primary analysis**										
CCA (n = 20)	10	50	0	0	8	40	0	0	2	10
MI (n = 14)	5	36	9	64	0	0	2	14	2	14
Other (n = 13)	10	77	2	15	1	8	0	0	1	8

*Note*. % = row percentages; CCA = complete‐case analysis; MAR = assuming data missing at random; MI = multiple imputation; MNAR = assuming data missing not at random. Total may be more than 100% as some studies conducted more than one sensitivity analysis.

a Other methods used for sensitivity analysis include last observation carried forward (*n* = 1), regression imputation (*n* = 1), adjusting for baseline predictors of missingness (*n* = 1), imputing by average of observed values for that patient (*n* = 1), and an ad hoc hybrid method using multiple and mean imputation (*n* = 1).

Only two studies (4%) conducted sensitivity analyses, assuming data could be MNAR. In both studies, values imputed under a standard MI were modified to incorporate possible departures from the MAR assumption for both the cost and effectiveness data using a simplified pattern‐mixture model approach (Faria et al., 2014; Leurent et al., 2018). The studies then discussed the plausibility of these departures from MAR and their implications for the cost‐effectiveness inferences.

### 3.5 Recommendations criteria

**Figure 3:**
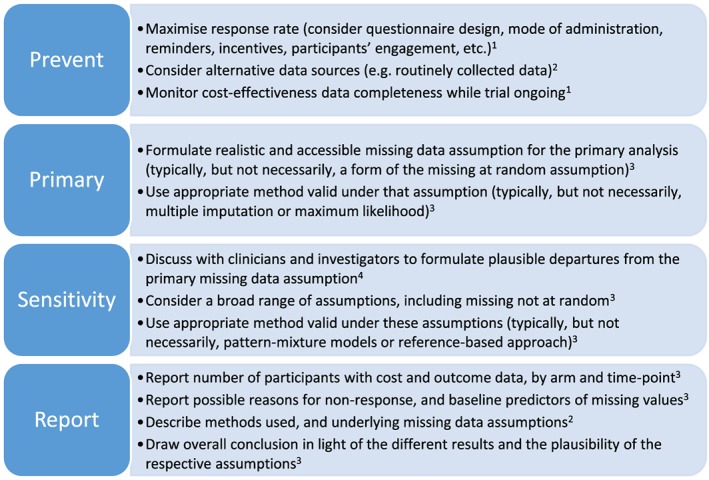
Recommendations for improving handling of missing data in trial‐based cost‐effectiveness analysis. References: 1, Little et al., 2012; 2, Noble et al., 2012; 3, Faria et al., 2014; and 4, Carpenter and Kenward 2007 [Colour figure can be viewed at http://wileyonlinelibrary.com]

Table 5 reports the number of studies that reported evidence of following the recommendations from Figure 3 (see Section 4). Most studies reported being aware of the risk of missing data, for example, by taking active steps to reduce them (*n* = 35, 74%). In addition, almost two‐thirds of the studies (*n* = 29, 62%) reported the breakdown of missing data by arm, time point, and endpoint. Only about one‐third of the studies have clearly reported the reasons for the missing data (*n* = 16, 34%) and the approach used for handling the missing data and its underlying assumptions (*n* = 17, 36%). Only one study (2%) appropriately discussed the implications of missing data in their cost‐effectiveness conclusions.

**Table 5:** Review of indicators based on recommendations criteria (*n* = 47)

Criterion^a^	Met^b^		Not met		Unclear	
	*n*	%	*n*	%	*n*	%
**Prevent**						
A1. Maximise response rate	35	74	12	26	0	0
A2. Alternative data sources	10	21	37	79	0	0
A3. Monitor completeness	17	36	30	64	0	0
**Primary**						
B1. Assumption for primary analysis	17	36	27	57	3	6
B2. Appropriate primary method	17	36	27	57	3	6
**Sensitivity**						
C1. Discuss departures from the primary assumption	0	0	47	100	0	0
C2. Consider broad range of assumptions	2	4	45	96	0	0
C3. Method valid under these assumptions	2	4	45	96	0	0
**Report**						
D1. Missing data by endpoint, arm, and time point	29	62	18	38	0	0
D2. Discuss reasons for missing data	16	34	31	66	0	0
D3. Describe methods used and assumptions	17	36	30	64	0	0
D4. Conclusions in light of missing data	1	2	46	98	0	0

a See Figure 3 and Appendix B for definition of each criterion.

b Report demonstrates evidence of having followed this recommendation. *Not met* if the recommendation was not followed or not mentioned. *Unclear* if some suggestions the criteria may have been met but information not clear enough. See Appendix B for detailed definitions and methodology used.

## 4 DISCUSSION

### 4.1 Summary of findings

Missing data remain ubiquitous in trial‐based CEA. The median proportion of participants with complete cost‐effectiveness data was only 63%. This reflects the typical challenges faced by CEA of randomised controlled trials, which often rely on patient questionnaires to collect key resource use and health outcome data. Despite best efforts to ensure completeness, a significant proportion of nonresponse is likely. This is consistent with other reviews, which also found no reduction of the extent of missing data in trials over time (Bell et al., 2014).

CCA remains the most commonly used approach for handling missing data in trial‐based CEA, in contrast to recommendations. This approach makes the restrictive assumption that, given the variables in the analysis model, the distributions of the outcome data are the same, whether or not those outcome data are observed. This approach is also problematic because it can result in a loss in precision, as it discards participants who have partially complete data postrandomisation and who can provide important information to the analysis. Other unsatisfactory approaches based on unrealistic assumptions, such as last observation carried forward and single imputation, are also occasionally used.

MI (Rubin, 1987) assuming MAR has been widely recommended for CEA (Briggs et al., 2003; Burton et al., 2007; Faria et al., 2014; Marshall et al., 2009), allowing for baseline variables and postrandomisation data not in the primary analysis to be used for the imputation. It seems to be now more commonly used, with around half of the studies using MI for at least one of their analyses (up to 74% in 2015). Around one‐third of the studies used MI for their primary CEA, which is higher than seen in primary clinical outcome analyses (8%; Bell et al., 2014).

On the other hand, sensitivity analyses to missing data remain clearly insufficient. Only two studies (4%) conducted comprehensive sensitivity analyses and assessed whether the study's conclusions were sensitive to departures from the MAR assumption (i.e., possible MNAR mechanisms). Half of the studies did not conduct any sensitivity analysis regarding the missing data. The remaining studies performed some sort of sensitivity analyses, but usually consisting of simple variations from the primary analysis, such as reporting CCA results in addition to MI. This may be more for completeness than proper missing data sensitivity analyses. For example, if MI is used for the primary analysis (having assumed that MAR is the realistic primary missing data assumption), a sensitivity analysis that involves CCA will make stronger missing data assumptions.

### 4.2 Strengths and limitations

Our review follows naturally from the review of Noble et al. (2012) and gives an update of the state of play after the publication of several key guidelines. Our review, however, differs in scope and methods and cannot be directly compared with the results of Noble et al. One of the key strengths of this review is that HTA comprehensive reports allowed us to obtain a more complete picture of the missing data and the methods used to tackle it. HTA monographs are published alongside more succinct peer‐reviewed papers in specialist medical journals, and they are often seen as the “gold‐standard” for trial‐based CEA in the UK. It seems therefore reasonable to assume that these are representative of typical practice in CEA. This review is, to our knowledge, the first to look at completeness of both cost and effectiveness data. A limitation is the use of a single‐indicator “proportion of complete cases” to capture the extent of the missing data issue. This is however a clearly defined indicator and allows comparison with other reviews. The “recommendations indicators” also focused on the information reported in the study, not necessarily what might have been done in practice.

### 4.3 Recommendations

A list of recommendations to address missing data in trial‐based CEA is presented in Figure 3. Trial‐based CEA are prone to missing data, and it is important that analysts take active steps at the design and data‐collection stages to limit their extent (Bernhard et al., 2006; Brueton et al., 2013; National Research Council, 2010). Resource use questionnaires should be designed in a user‐friendly way, and their completion encouraged during follow‐up visits, possibly supported by a researcher (Mercieca‐Bebber et al., 2016; National Research Council, 2010). Alternative sources should also be considered to minimise missing information, for example, administrative data or electronic health records (Franklin & Thorn, 2018; Noble et al., 2012).

For any study with missing data, clear reporting of the issue is required. Ideally, the study should report details of the pattern of missing data (Faria et al., 2014), possibly as an appendix. At a minimum, CEA studies should report for each analysis the number of participants included by trial arm, as recommended in the Consolidated Standards of Reporting Trials guidelines (Noble et al., 2012; Schulz et al., 2010).

Although CCA may be justifiable in some circumstances, the choice of CCA for the primary analysis approach appears difficult to justify in the presence of repeated measurements, because the loss of power (by discarding all patients with any missing values) across the different time points tends to be large. Other approaches valid under more plausible MAR assumptions and making use of all the observed data, such as MI (Rubin, 1987); likelihood‐based repeated measures models (Faria et al., 2014; Verbeke, Fieuws, Molenberghs, & Davidian, 2014); or Bayesian models (Ades et al., 2006), should be considered. In particular, MI has been increasingly used in CEA, and further guidance to support an appropriate use in this context is warranted.

An area with clear room for improvement is the conduct of sensitivity analyses. This review found that many studies used CCA for the primary analysis and MI as a sensitivity analysis, or vice‐versa, and concluded that the results were robust to missing data. This is misleading because both of these methods rely on the assumption that the missingness is independent of the unobserved data. Although the MAR assumption provides a sensible starting point, it is not possible to determine the true missing‐data mechanism from the observed data. Studies should therefore assess whether their conclusions are sensitive to possible departures from that assumption (National Research Council, 2010; Committee for Medicinal Products for Human Use (CHMP), 2011; Faria et al., 2014). Several approaches have been suggested to conduct analyses under MNAR assumptions. Selection models express how the probability of being missing is related to the value itself. Pattern‐mixture models, on the other hand, capture how missing data could differ from the observed (Molenberghs et al., 2014; Ratitch, O'Kelly, & Tosiello, 2013). Pattern‐mixture models appear attractive because they frame the departure from MAR in a way that can be more readily understood by clinical experts and decision makers and can be used with standard analysis methods such as MI (Carpenter & Kenward, 2012; Ratitch et al., 2013). MNAR modelling can be challenging, but accessible approaches have also been proposed (Faria et al., 2014; Leurent et al., 2018). Further developments are still needed to use these methods in the CEA context and to provide the analytical tools and practical guidance to implement them in practice.

## 5 CONCLUSION

Missing data can be an important source of bias and uncertainty, and it is imperative that this issue is appropriately recognised and addressed to help ensure that CEA studies provide sound evidence for healthcare decision making. Over the last decade, there have been some welcome improvements in handling missing data in trial‐based CEA. In particular, more attention has been devoted to assessing the reasons for the missing data and adopting methods (e.g., MI) that can incorporate those in the analysis. However, there is substantial room for improvement. Firstly, more efforts are needed to reduce missing data. Secondly, the extent and patterns of missing data should be more clearly reported. Thirdly, the primary analysis should consider methods that make contextually plausible assumptions rather than resort automatically to CCA. Lastly, sensitivity analyses to assess the robustness of the study's results to potential MNAR mechanisms should be conducted.

## CONFLICT OF INTEREST

The authors have no conflict of interest.

## APPENDIX A DETAILS OF STUDIES SELECTION

### A.1 PubMed search criteria and results

Search criteria:

(“Health Technol Assess”[Journal])

AND (“2015/01/01”[Date ‐ Publication]: “2015/12/31”[Date ‐ Publication])

AND (“randomised”[Title] OR “randomized”[Title] OR “trial”[Title])

AND (“economic”[Title/Abstract] OR “cost*”[Title/Abstract])

NOT (“pilot”[Title] OR “feasibility”[Title])

Number of studies:

**Table:**

Search	Query	Items found
4	Search (“health Technol assess”[journal]) AND (“2013/01/01”[date ‐ publication] : “2015/12/31”[date ‐ publication]) AND (“randomised”[title] OR “randomised”[title] OR “trial”[title]) AND (“economic”[title/abstract] OR “cost*”[title/abstract]) NOT (“pilot”[title] OR “feasibility”[title])	65
3	Search (“Health Technol Assess”[Journal]) AND (“2013/01/01”[Date ‐ Publication] : “2015/12/31”[Date ‐ Publication]) AND (“randomised”[title] OR “randomized”[Title] OR “trial”[Title]) AND (“economic”[Title/Abstract] OR “cost*”[Title/Abstract])	74
2	Search (“Health Technol Assess”[Journal]) AND (“2013/01/01”[Date ‐ Publication] : “2015/12/31”[Date ‐ Publication]) AND (“randomised”[Title] OR “randomized”[Title] OR “trial”[Title])	91
1	Search (“Health Technol Assess”[Journal]) AND (“2013/01/01”[Date ‐ Publication] : “2015/12/31”[Date ‐ Publication])	236

### A.2 Included studies

Allen, S. , Wareham, K. , Wang, D. , Bradley, C. , Sewell, B. , Hutchings, H. , … Phillips, C. (2013). A high‐dose preparation of lactobacilli and bifidobacteria in the prevention of antibiotic‐associated and Clostridium difficile diarrhoea in older people admitted to hospital: A multicentre, randomised, double‐blind, placebo‐controlled, parallel arm trial. Health Technology Assessment, 17(57), 1–140. http://doi.org/10.3310/hta17570 10.3310/hta17570PMC478164724309198

Ashby, R. L. , Gabe, R. , Ali, S. , Saramago, P. , Chuang, L.‐H. , Adderley, U. , … Torgerson, D. J. (2014). VenUS IV (Venous leg Ulcer Study IV) – compression hosiery compared with compression bandaging in the treatment of venous leg ulcers: A randomised controlled trial, mixed‐treatment comparison and decision‐analytic model. Health Technology Assessment, 18(57), 1–294. http://doi.org/10.3310/hta18570 10.3310/hta18570PMC478120225242076

Banerjee, S. , Hellier, J. , Romeo, R. , Dewey, M. , Knapp, M. , Ballard, C. , … Burns, A. (2013). Study of the use of antidepressants for depression in dementia: the HTA‐SADD trial—A multicentre, randomised, double‐blind, placebo‐controlled trial of the clinical effectiveness and cost‐effectiveness of sertraline and mirtazapine. Health Technology Assessment (Winchester, England), 17(7), 1–166. http://doi.org/10.3310/hta17070 10.3310/hta17070PMC478281123438937

Bedson, E. , Bell, D. , Carr, D. , Carter, B. , Hughes, D. , Jorgensen, A. , … Williams, N. (2014). Folate Augmentation of Treatment – Evaluation for Depression (FolATED): Randomised trial and economic evaluation. Health Technology Assessment, 18(48), 1–160. http://doi.org/10.3310/hta18480 10.3310/hta18480PMC478099125052890

Blyth, A. , Maskrey, V. , Notley, C. , Barton, G. R. , Brown, T. J. , Aveyard, P. , … Song, F. (2015). Effectiveness and economic evaluation of self‐help educational materials for the prevention of smoking relapse: Randomised controlled trial. Health Technology Assessment (Winchester, England), 19(59), 1–70, v–vi. http://doi.org/10.3310/hta19590 10.3310/hta19590PMC478099426218035

Brittenden, J. , Cotton, S. C. , Elders, A. , Tassie, E. , Scotland, G. , Ramsay, C. R. , … Campbell, M. K. (2015). Clinical effectiveness and cost‐effectiveness of foam sclerotherapy, endovenous laser ablation and surgery for varicose veins: Results from the Comparison of LAser, Surgery and foam Sclerotherapy (CLASS) randomised controlled trial. Health Technology Assessment (Winchester, England), 19(27), 1–342. http://doi.org/10.3310/hta19270 10.3310/hta19270PMC478154425858333

Carr, A. J. , Cooper, C. D. , Campbell, M. K. , Rees, J. L. , Moser, J. , Beard, D. J. , … Ramsay, C. R. (2015). Clinical effectiveness and cost‐effectiveness of open and arthroscopic rotator cuff repair [the UK Rotator Cuff Surgery (UKUFF) randomised trial]. Health Technology Assessment (Winchester, England), 19(80), 1–218. http://doi.org/10.3310/hta19800 10.3310/hta19800PMC478104126463717

Chakravarthy, U. , Harding, S. P. , Rogers, C. A. , Downes, S. , Lotery, A. J. , Dakin, H. A. , … Reeves, B. C. (2015). A randomised controlled trial to assess the clinical effectiveness and cost‐effectiveness of alternative treatments to Inhibit VEGF in Age‐related choroidal Neovascularisation (IVAN). Health Technology Assessment, 19(78), 1–298. http://doi.org/10.3310/hta19780 10.3310/hta19780PMC478141626445075

Clark, T. J. , Middleton, L. J. , Cooper, N. A. , Diwakar, L. , Denny, E. , Smith, P. , … Daniels, J. P. (2015). A randomised controlled trial of Outpatient versus inpatient Polyp Treatment (OPT) for abnormal uterine bleeding. Health Technology Assessment, 19(61), 1–194. http://doi.org/10.3310/hta19610 10.3310/hta19610PMC478138326240949

Cooper, S. , Lewis, S. , Thornton, J. G. , Marlow, N. , Watts, K. , Britton, J. , … Coleman, T. (2014). The SNAP trial: a randomised placebo‐controlled trial of nicotine replacement therapy in pregnancy – clinical effectiveness and safety until 2 years after delivery, with economic evaluation. Health Technology Assessment, 18(54), 1–128. http://doi.org/10.3310/hta18540 10.3310/hta18540PMC478281225158081

Costa, M. L. , Achten, J. , Plant, C. , Parsons, N. R. , Rangan, A. , Tubeuf, S. , … Lamb, S. E. (2015). UK DRAFFT: A randomised controlled trial of percutaneous fixation with Kirschner wires versus volar locking‐plate fixation in the treatment of adult patients with a dorsally displaced fracture of the distal radius. Health Technology Assessment, 19(17), 1–124. http://doi.org/10.3310/hta19170 10.3310/hta19170PMC478114925716883

Crawford, M. J. , Sanatinia, R. , Barrett, B. , Byford, S. , Dean, M. , Green, J. , … Ward, H. (2014). The clinical effectiveness and cost‐effectiveness of brief intervention for excessive alcohol consumption among people attending sexual health clinics: A randomised controlled trial (SHEAR). Health Technology Assessment, 18(30), 1–48. http://doi.org/10.3310/hta18300 10.3310/hta18300PMC478150024813652

Creswell, C. , Cruddace, S. , Gerry, S. , Gitau, R. , McIntosh, E. , Mollison, J. , … Cooper, P. J. (2015). Treatment of childhood anxiety disorder in the context of maternal anxiety disorder: A randomised controlled trial and economic analysis. Health Technology Assessment, 19(38), 1–184. http://doi.org/10.3310/hta19380 10.3310/hta19380PMC478133026004142

Cunningham, S. , Rodriguez, A. , Boyd, K. A. , McIntosh, E. , & Lewis, S. C. (2015). Bronchiolitis of Infancy Discharge Study (BIDS): A multicentre, parallel‐group, double‐blind, randomised controlled, equivalence trial with economic evaluation. Health Technology Assessment, 19(71), 1–172. http://doi.org/10.3310/hta19710 10.3310/hta19710PMC478097526364905

Dennis, M. , Sandercock, P. , Graham, C. , & Forbes, J. (2015). The Clots in Legs Or sTockings after Stroke (CLOTS) 3 trial: A randomised controlled trial to determine whether or not intermittent pneumatic compression reduces the risk of post‐stroke deep vein thrombosis and to estimate its cost‐effectiveness. Health Technology Assessment, 19(76), 1–90. http://doi.org/10.3310/hta19760 10.3310/hta19760PMC478281426418530

Everard, M. L. , Hind, D. , Ugonna, K. , Freeman, J. , Bradburn, M. , Dixon, S. , … Cross, E. (2015). Saline in Acute Bronchiolitis RCT and Economic evaluation: Hypertonic saline in acute bronchiolitis – randomised controlled trial and systematic review. Health Technology Assessment, 19(66), 1–130. http://doi.org/10.3310/hta19660 10.3310/hta19660PMC478152926295732

Forster, A. , Dickerson, J. , Young, J. , Patel, A. , Kalra, L. , Nixon, J. , … Farrin, A. (2013). A cluster randomised controlled trial and economic evaluation of a structured training programme for caregivers of inpatients after stroke: The TRACS trial. Health Technology Assessment, 17(46), 1–216. http://doi.org/10.3310/hta17460 10.3310/hta17460PMC496781124153026

Gates, S. , Perkins, G. , Lamb, S. , Kelly, C. , Thickett, D. , Young, J. , … Gao Smith, F. (2013). Beta‐Agonist Lung injury TrIal‐2 (BALTI‐2): A multicentre, randomised, double‐blind, placebo‐controlled trial and economic evaluation of intravenous infusion of salbutamol versus placebo in patients with acute respiratory distress syndrome. Health Technology Assessment, 17(38), v–vi, 1–87. http://doi.org/10.3310/hta17380 10.3310/hta17380PMC478153224028755

Goyder, E. , Hind, D. , Breckon, J. , Dimairo, M. , Minton, J. , Everson‐Hock, E. , … Cooper, C. (2014). A randomised controlled trial and cost‐effectiveness evaluation of “booster” interventions to sustain increases in physical activity in middle‐aged adults in deprived urban neighbourhoods. Health Technology Assessment, 18(13), 1–210. http://doi.org/10.3310/hta18130 10.3310/hta18130PMC478119324571932

Grant, A. , Boachie, C. , Cotton, S. , Faria, R. , Bojke, L. , Epstein, D. , … Campbell, M. (2013). Clinical and economic evaluation of laparoscopic surgery compared with medical management for gastro‐oesophageal reflux disease: 5‐year follow‐up of multicentre randomised trial (the REFLUX trial). Health Technology Assessment, 17(22), 1–167. http://doi.org/10.3310/hta17220 10.3310/hta17220PMC478127623742987

Gupta, J. K. , Daniels, J. P. , Middleton, L. J. , Pattison, H. M. , Prileszky, G. , Roberts, T. E. , … Kai, J. (2015). A randomised controlled trial of the clinical effectiveness and cost‐effectiveness of the levonorgestrel‐releasing intrauterine system in primary care against standard treatment for menorrhagia: The ECLIPSE trial. Health Technology Assessment, 19(88), 1–118. http://doi.org/10.3310/hta19880 10.3310/hta19880PMC478134526507206

Halligan, S. , Dadswell, E. , Wooldrage, K. , Wardle, J. , von Wagner, C. , Lilford, R. , … Atkin, W. (2015). Computed tomographic colonography compared with colonoscopy or barium enema for diagnosis of colorectal cancer in older symptomatic patients: Two multicentre randomised trials with economic evaluation (the SIGGAR trials). Health Technology Assessment, 19(54), 1–134. http://doi.org/10.3310/hta19540 10.3310/hta19540PMC478128426198205

Handoll, H. , Brealey, S. , Rangan, A. , Keding, A. , Corbacho, B. , Jefferson, L. , … Torgerson, D. (2015). The ProFHER (PROximal Fracture of the Humerus: Evaluation by Randomisation) trial – a pragmatic multicentre randomised controlled trial evaluating the clinical effectiveness and cost‐effectiveness of surgical compared with non‐surgical treatment for proxi. Health Technology Assessment, 19(24), 1–280. http://doi.org/10.3310/hta19240 10.3310/hta19240PMC478105225822598

Iliffe, S. , Kendrick, D. , Morris, R. , Masud, T. , Gage, H. , Skelton, D. , … Belcher, C. (2014). Multicentre cluster randomised trial comparing a community group exercise programme and home‐based exercise with usual care for people aged 65 years and over in primary care. Health Technology Assessment, 18(49), 1–106. http://doi.org/10.3310/hta18490 10.3310/hta18490PMC478114425098959

Kuyken, W. , Hayes, R. , Barrett, B. , Byng, R. , Dalgleish, T. , Kessler, D. , … Byford, S. (2015). The effectiveness and cost‐effectiveness of mindfulness‐based cognitive therapy compared with maintenance antidepressant treatment in the prevention of depressive relapse/recurrence: Results of a randomised controlled trial (the PREVENT study). Health Technology Assessment, 19(73), 1–124. http://doi.org/10.3310/hta19730 10.3310/hta19730PMC478144826379122

Lall, R. , Hamilton, P. , Young, D. , Hulme, C. , Hall, P. , Shah, S. , … Lamb, S. (2015). A randomised controlled trial and cost‐effectiveness analysis of high‐frequency oscillatory ventilation against conventional artificial ventilation for adults with acute respiratory distress syndrome. The OSCAR (OSCillation in ARDS) study. Health Technology Assessment, 19(23), 1–178. http://doi.org/10.3310/hta19230 10.3310/hta19230PMC478159025800686

Lenney, W. , McKay, A. J. , Tudur Smith, C. , Williamson, P. R. , James, M. , Price, D. , & MASCOT Study Group . (2013). Management of Asthma in School age Children On Therapy (MASCOT): A randomised, double‐blind, placebo‐controlled, parallel study of efficacy and safety. Health Technology Assessment (Winchester, England), 17(4), 1–218. http://doi.org/10.3310/hta17040 10.3310/hta17040PMC478121923380178

Little, P. , Hobbs, F. R. , Moore, M. , Mant, D. , Williamson, I. , McNulty, C. , … Mullee, M. (2014). PRImary care Streptococcal Management (PRISM) study: in vitro study, diagnostic cohorts and a pragmatic adaptive randomised controlled trial with nested qualitative study and cost‐effectiveness study. Health Technology Assessment, 18(6), vii–xxv, 1–101. http://doi.org/10.3310/hta18060 10.3310/hta18060PMC478154524467988

Littlewood, E. , Duarte, A. , Hewitt, C. , Knowles, S. , Palmer, S. , Walker, S. , … Gilbody, S. (2015). A randomised controlled trial of computerised cognitive behaviour therapy for the treatment of depression in primary care: The Randomised Evaluation of the Effectiveness and Acceptability of Computerised Therapy (REEACT) trial. Health Technology Assessment, 19(101), 1–174. http://doi.org/10.3310/hta191010 10.3310/hta191010PMC478116526685904

Livingston, G. , Barber, J. , Rapaport, P. , Knapp, M. , Griffin, M. , Romeo, R. , … Cooper, C. (2014). START (STrAtegies for RelaTives) study: A pragmatic randomised controlled trial to determine the clinical effectiveness and cost‐effectiveness of a manual‐based coping strategy programme in promoting the mental health of carers of people with dementia. Health Technology Assessment, 18(61), 1–242. http://doi.org/10.3310/hta18610 10.3310/hta18610PMC478146725300037

Logan, P. A. , Armstrong, S. , Avery, T. J. , Barer, D. , Barton, G. R. , Darby, J. , … Leighton, M. P. (2014). Rehabilitation aimed at improving outdoor mobility for people after stroke: a multicentre randomised controlled study (the Getting out of the House Study). Health Technology Assessment, 18(29), vii–viii, 1–113. http://doi.org/10.3310/hta18290 10.3310/hta18290PMC478105624806825

McMillan, A. , Bratton, D. J. , Faria, R. , Laskawiec‐Szkonter, M. , Griffin, S. , Davies, R. J. , … Morrell, M. J. (2015). A multicentre randomised controlled trial and economic evaluation of continuous positive airway pressure for the treatment of obstructive sleep apnoea syndrome in older people: PREDICT. Health Technology Assessment, 19(40), 1–188. http://doi.org/10.3310/hta19400 10.3310/hta19400PMC478094826063688

Molassiotis, A. , Russell, W. , Hughes, J. , Breckons, M. , Lloyd‐Williams, M. , Richardson, J. , … Ryder, W. (2013). The effectiveness and cost‐effectiveness of acupressure for the control and management of chemotherapy‐related acute and delayed nausea: Assessment of Nausea in Chemotherapy Research (ANCHoR), a randomised controlled trial. Health Technology Assessment, 17(26), 1–114. http://doi.org/10.3310/hta17260 10.3310/hta17260PMC478125623803562

Morris, R. , Malin, G. , Quinlan‐Jones, E. , Middleton, L. , Diwakar, L. , Hemming, K. , … Kilby, M. (2013). The Percutaneous shunting in Lower Urinary Tract Obstruction (PLUTO) study and randomised controlled trial: evaluation of the effectiveness, cost‐effectiveness and acceptability of percutaneous vesicoamniotic shunting for lower urinary tract obstruction. Health Technology Assessment, 17(59), 1–232. http://doi.org/10.3310/hta17590 10.3310/hta17590PMC478139324331029

Mouncey, P. R. , Osborn, T. M. , Power, G. S. , Harrison, D. A. , Sadique, M. Z. , Grieve, R. D. , … Rowan, K. M. (2015). Protocolised Management In Sepsis (ProMISe): A multicentre randomised controlled trial of the clinical effectiveness and cost‐effectiveness of early, goal‐directed, protocolised resuscitation for emerging septic shock. Health Technology Assessment, 19(97), 1–150. http://doi.org/10.3310/hta19970 10.3310/hta19970PMC478148226597979

Murray, D. W. , MacLennan, G. S. , Breeman, S. , Dakin, H. A. , Johnston, L. , Campbell, M. K. , … Grant, A. M. (2014). A randomised controlled trial of the clinical effectiveness and cost‐effectiveness of different knee prostheses: The Knee Arthroplasty Trial (KAT). Health Technology Assessment, 18(19), 1–235, vii–viii. http://doi.org/10.3310/hta18190 10.3310/hta18190PMC478156524679222

Nicholson, K. G. , Abrams, K. R. , Batham, S. , Medina, M. J. , Warren, F. C. , Barer, M. , … Zambon, M. (2014). Randomised controlled trial and health economic evaluation of the impact of diagnostic testing for influenza, respiratory syncytial virus and Streptococcus pneumoniae infection on the management of acute admissions in the elderly and high‐risk 18‐ to 64‐y. Health Technology Assessment, 18(36), 1–274, vii–viii. http://doi.org/10.3310/hta18360 10.3310/hta18360PMC478160524875092

Orgeta, V. , Leung, P. , Yates, L. , Kang, S. , Hoare, Z. , Henderson, C. , … Orrell, M. (2015). Individual cognitive stimulation therapy for dementia: a clinical effectiveness and cost‐effectiveness pragmatic, multicentre, randomised controlled trial. Health Technology Assessment, 19(64), 1–108. http://doi.org/10.3310/hta19640 10.3310/hta19640PMC478145426292178

Pickard, R. , Starr, K. , MacLennan, G. , Kilonzo, M. , Lam, T. , Thomas, R. , … McClinton, S. (2015). Use of drug therapy in the management of symptomatic ureteric stones in hospitalised adults: A multicentre, placebo‐controlled, randomised controlled trial and cost‐effectiveness analysis of a calcium channel blocker (nifedipine) and an alpha‐blocker (tam. Health Technology Assessment (Winchester, England)), 19(63), vii–viii, 1–171. http://doi.org/10.3310/hta19630 10.3310/hta19630PMC478161626244520

Powell, C. , Kolamunnage‐Dona, R. , Lowe, J. , Boland, A. , Petrou, S. , Doull, I. , … Williamson, P. (2013). MAGNEsium Trial In Children (MAGNETIC): A randomised, placebo‐controlled trial and economic evaluation of nebulised magnesium sulphate in acute severe asthma in children. Health Technology Assessment, 17(45), v–vi, 1–216. http://doi.org/10.3310/hta17450 10.3310/hta17450PMC478138024144222

Russell, I. , Edwards, R. , Gliddon, A. , Ingledew, D. , Russell, D. , Whitaker, R. , … Park, K. (2013). Cancer of Oesophagus or Gastricus – New Assessment of Technology of Endosonography (COGNATE): report of pragmatic randomised trial. Health Technology Assessment, 17(39), 1–170. http://doi.org/10.3310/hta17390 10.3310/hta17390PMC478142924034150

Salisbury, C. , Foster, N. , Hopper, C. , Bishop, A. , Hollinghurst, S. , Coast, J. , … Montgomery, A. (2013). A pragmatic randomised controlled trial of the effectiveness and cost‐effectiveness of “PhysioDirect” telephone assessment and advice services for physiotherapy. Health Technology Assessment, 17(02), 1–157, v–vi. http://doi.org/10.3310/hta17020 10.3310/hta17020PMC478092123356839

Scott, D. L. , Ibrahim, F. , Farewell, V. , O'Keeffe, A. G. , Ma, M. , Walker, D. , … Kingsley, G. (2014). Randomised controlled trial of Tumour necrosis factor inhibitors Against Combination Intensive Therapy with conventional disease‐modifying antirheumatic drugs in established rheumatoid arthritis: The TACIT trial and associated systematic reviews. Health Technology Assessment, 18(66), 1–164. http://doi.org/10.3310/hta18660 10.3310/hta18660PMC478095825351370

Sharples, L. , Glover, M. , Clutterbuck‐James, A. , Bennett, M. , Jordan, J. , Chadwick, R. , … Quinnell, T. (2014). Clinical effectiveness and cost‐effectiveness results from the randomised controlled Trial of Oral Mandibular Advancement Devices for Obstructive sleep apnoea–hypopnoea (TOMADO) and long‐term economic analysis of oral devices and continuous positive airwa. Health Technology Assessment, 18(67), 1–296. http://doi.org/10.3310/hta18670 10.3310/hta18670PMC478112125359435

Stallard, P. , Phillips, R. , Montgomery, A. , Spears, M. , Anderson, R. , Taylor, J. , … Sayal, K. (2013). A cluster randomised controlled trial to determine the clinical effectiveness and cost‐effectiveness of classroom‐based cognitive–behavioural therapy (CBT) in reducing symptoms of depression in high‐risk adolescents. Health Technology Assessment, 17(47), vii–xvii, 1–109. http://doi.org/10.3310/hta17470 10.3310/hta17470PMC478120724172024

Thursz, M. , Forrest, E. , Roderick, P. , Day, C. , Austin, A. , O'Grady, J. , … Ternent, L. (2015). The clinical effectiveness and cost‐effectiveness of STeroids Or Pentoxifylline for Alcoholic Hepatitis (STOPAH): A 2 × 2 factorial randomised controlled trial. Health Technology Assessment, 19(102), 1–104. http://doi.org/10.3310/hta191020 10.3310/hta191020PMC478110326691209

Underwood, M. , Lamb, S. , Eldridge, S. , Sheehan, B. , Slowther, A. , Spencer, A. , … Taylor, S. (2013). Exercise for depression in care home residents: a randomised controlled trial with cost‐effectiveness analysis (OPERA). Health Technology Assessment, 17(18), 1–281. http://doi.org/10.3310/hta17180 10.3310/hta17180PMC478149823632142

Ussher, M. , Lewis, S. , Aveyard, P. , Manyonda, I. , West, R. , Lewis, B. , … Coleman, T. (2015). The London Exercise And Pregnant smokers (LEAP) trial: A randomised controlled trial of physical activity for smoking cessation in pregnancy with an economic evaluation. Health Technology Assessment, 19(84), 1–136. http://doi.org/10.3310/hta19840 10.3310/hta19840PMC478110126491878

Watson, J. , Crosby, H. , Dale, V. , Tober, G. , Wu, Q. , Lang, J. , … Coulton, S. (2013). AESOPS: A randomised controlled trial of the clinical effectiveness and cost‐effectiveness of opportunistic screening and stepped care interventions for older hazardous alcohol users in primary care. Health Technology Assessment, 17(25), 1–158. http://doi.org/10.3310/hta17250 10.3310/hta17250PMC478094523796191

Wiles, N. , Thomas, L. , Abel, A. , Barnes, M. , Carroll, F. , Ridgway, N. , … Lewis, G. (2014). Clinical effectiveness and cost‐effectiveness of cognitive behavioural therapy as an adjunct to pharmacotherapy for treatment‐resistant depression in primary care: The CoBalT randomised controlled trial. Health Technology Assessment, 18(31), 1–167, vii–viii. http://doi.org/10.3310/hta18310 10.3310/hta18310PMC478119824824481

Williams, M. A. , Williamson, E. M. , Heine, P. J. , Nichols, V. , Glover, M. J. , Dritsaki, M. , … Lamb, S. E. (2015). Strengthening And stretching for Rheumatoid Arthritis of the Hand (SARAH). A randomised controlled trial and economic evaluation. Health Technology Assessment, 19(19), 1–222. http://doi.org/10.3310/hta19190 10.3310/hta19190PMC478089325748549

Wolf, A. , McKay, A. , Spowart, C. , Granville, H. , Boland, A. , Petrou, S. , … Gamble, C. (2014). Prospective multicentre randomised, double‐blind, equivalence study comparing clonidine and midazolam as intravenous sedative agents in critically ill children: The SLEEPS (Safety profiLe, Efficacy and Equivalence in Paediatric intensive care Sedation) st. Health Technology Assessment, 18(71), 1–212. http://doi.org/10.3310/hta18710 10.3310/hta18710PMC478131426099138

## APPENDIX B INDICATORS DEFINITION

### B.1 Primary indicators

**Table:**

Indicator	Definition	Notes
Proportion of complete cases	Proportion of randomised participants for whom all data were available for the primary cost‐effectiveness analysis	If the number of complete‐cases was not clearly reported, we estimated an “upper bound,” from information, such as the proportion of participants with complete cost, or effect, data. See definition of primary analysis below.
Proportion complete effectiveness data	Proportion of randomised participants for whom all effectiveness data were Available for the primary cost‐effectiveness analysis	Same as above
Proportion complete cost data	Proportion of randomised participants for whom all cost data were available for the primary cost‐effectiveness analysis	Same as above
Report exact number of complete cases	Whether the number of participants with complete cost and effectiveness data was clearly reported.	
More missing costs or effectiveness	Whether the proportion of complete cases differ between cost and effectiveness variable.	Considered “similar” when the proportion of complete cases was within 5% of each other.
Primary analysis method	Methods used to address missing data in the primary (base case) cost‐effectiveness analysis	When multiple effectiveness measures, time‐frames, or cost perspectives were reported, without a base‐case clearly defined, we considered the analysis based on quality‐adjusted life years (QALYs) over the longest within‐trial follow‐up period, from the NHS and social services cost perceptive.
Conducted a sensitivity analysis to missing data	Report results under more than one approach for addressing missing data	

### B.2 Secondary indicators: Derived from the recommendations list

#### B.2.1 Methods

Because these aspects could have been mentioned in multiple parts in the monograph, we used a systematic approach, looking for keywords and checking the most relevant paragraphs in the full report.

- Search in PDF: “Missing”; “Participation”; “Completion”; “Incomplete”; “Response”; “Non‐response”; “Monitor”; “MCAR”; “MAR”; “MNAR.”
- If did not find “steps to reduce missing data,” also check “reminder,” “incentive,” “telephone,” and “contact.”
- Then, check relevant paragraphs manually: data source for cost‐effectiveness data; beginning of CEA results; and CEA conclusions.

#### B.2.2 Answers

“Yes”: The recommendation was clearly mentioned, and the criteria therefore met.

“No”: The recommendation was not clearly mentioned or found. The recommendation may still have been followed but not reported (or at least not found with the above strategy).

“Unclear”: There was some suggestions the criteria may have been met but not enough information to be sure.

**Table:**

Recommendation	Indicator definition	Examples “yes”	Examples “no”	Notes
A1. Maximise response rate (consider questionnaire design, mode of administration, reminders, incentives, participants' engagement, etc.)	Mention taking steps to maximise response rate	Reminder, incentives, home/hospital visit, multiple attempts,	Mention response was maximised for clinical outcome but not reported for cost‐effectiveness endpoints	Can be for overall trial data if implicit includes cost or effect data. Except if steps are clearly for non‐CE variables only (e.g., primary outcome only).
A2. Consider alternative data sources (e.g., routinely collected data)	Mention that considered missing data issues when choosing appropriate source, OR mention more than one source used for a CE data.	Use of electronic health records or administrative data, e.g., hospital episode statistics were used to supplement trial's data, for example, about hospital admissions post‐randomisation (which might be otherwise missing).	Using routine data as a primary source: e.g., resource use taken primarily from administrative/hospital records.	
A3. Monitor cost‐effectiveness data completeness while trial ongoing	Mentioned monitoring data completeness while trial ongoing.	Data managers checked inconsistent and missing data (if not clear “while trial ongoing” but mention monitoring probably fine). Mention taking new steps to reduce MD (e.g., incentive) as realised lots of MD after trial started.	Mention data checks for inconsistencies, but no mention of checking missing data.	Can be for overall trial data. Except if monitoring clearly for non‐CE variables only (e.g., primary outcome only).
B1. Formulate realistic and accessible missing data assumption for the primary analysis (typically, but not necessarily, a form of the missing at random assumption)	Primary (base‐case) CEA based on reasonable missing data assumptions. (likely MAR, or alternative if well justified).	– Used MI for primary analysis ‐ well justified and clear alternative	– Hybrid method, except if clearly explain and justify underlying assumptions	
B2. Use appropriate method valid under that assumption (typically, but not necessarily, multiple imputation or maximum likelihood)	Use appropriate analysis method.	– MI for primary analysis ‐Bayesian under MAR ‐ well justified and clear alternative	– Use unadjusted CCA when reporting data are MAR.	
C1. Discuss with clinicians and investigators to formulate plausible departures from the primary missing data assumption		Conducted MNAR SA + mention elicitation.	Did not conduct MNAR SA	
C2. Consider a broad range of assumptions, including missing not at random mechanisms		Conducted MNAR SA	Did not conduct MNAR SA	
C3. Use appropriate method valid under these assumptions (typically, but not necessarily, pattern‐mixture models or reference‐based approach)		Conducted MNAR SA, and used an appropriate method (PMM, etc.).	Did not conduct MNAR SA	
D1. Report number of participants with cost and outcome data, by arm and time‐point	Report number (or %) of complete or missing data. Split at least by effectiveness vs. cost, time point (when applicable), and arm		Reported missing data by endpoint and arm, but not by time point.	Do not have to be all at the same time (split by endpoint + time + arm), can be three separate table/texts.
D2. Report possible reasons for non‐response, and baseline predictors of missing values	Mention something about main reason for the missing data, OR Explore factors associated with it.	Comment on why missing data (e.g., “because patients were too ill”). Or explore baseline factors associated with missingness	No mention of reasons for MD in the CE section.	Have to be specific to the CE missing data, or clearly mentioning something like “reasons for MD are discussed in clinical analysis section …”
D3. Describe methods used, and underlying missing data assumptions	Clearly state the method used to address missing data, AND the underlying assumption.		No report of missing data assumption or method used	
Draw overall conclusion in light of the different results and the plausibility of the respective assumptions	Conduct sensitivity analyses, and interpret results appropriately.	Did MNAR SA and appropriate conclusion.	– Did not conduct sensitivity analyses – Conducted sensitivity analyses, but no comment/conclusion – Did MI and CC and only say “results did not change/robust to missing data”	
